# Characterizing Pedestrian Network from Segmented 3D Point Clouds for Accessibility Assessment: A Virtual Robotic Approach

**DOI:** 10.3390/s26072172

**Published:** 2026-03-31

**Authors:** Ali Ahmadi, Mir Abolfazl Mostafavi, Ernesto Morales, Nouri Sabo

**Affiliations:** 1 Center for Research in Geospatial Data and Intelligence (CRDIG), Department of Geomatics Sciences, Université Laval, 1055, Avenue du Séminaire, Quebec City, QC G1V 0A6, Canada; mir-abolfazl.mostafavi@scg.ulaval.ca; 2 Centre for Interdisciplinary Research in Rehabilitation and Social Integration (Cirris), Quebec City, QC G1M 2S8, Canada; ernesto.morales@fmed.ulaval.ca; 3 School of Rehabilitation Sciences, Faculty of Medicine, Laval University, Quebec City, QC G1V 0A6, Canada; 4 Canada Centre for Mapping and Earth Observation, Sherbrooke, QC J1H 4G9, Canada; nouri.sabo@nrcan-rncan.gc.ca

**Keywords:** accessibility assessment, robotic, Tangent Bug, environmental factors, characterization, 3D point cloud

## Abstract

This study introduces a novel virtual robotic approach for automated characterization of pedestrian network accessibility from semantically segmented 3D LiDAR point clouds. With approximately 8 million Canadians living with disabilities, scalable accessibility assessment methods are critical. The proposed methodology integrates a Tangent Bug navigation algorithm—extended from 2D to 3D point cloud environments—with a triangular virtual robot grounded in ADA and IBC accessibility standards. The robot navigates classified point cloud data to simultaneously extract related parameters per step including those related to the accessibility assessment, including running slope, cross-slope, path width, surface type, and step height, aligned with the Measure of Environmental Accessibility (MEA) framework. Unlike existing approaches, the method characterizes not only formal sidewalk segments but also the critical transitional linkages between building entrances and the pedestrian network. Rather than evaluating features against fixed binary thresholds, it records continuous raw measurements enabling personalized accessibility assessment tailored to individual user profiles. Quantitative validation demonstrates high accuracy for path width (NRMSE = 2.71%) and reliable slope tracking. The proposed approach is faster, more cost-effective, and more comprehensive than traditional manual methods, and its segment-independent architecture makes it well-suited for future city-scale deployment.

## 1. Introduction

Social participation poses a significant challenge for individuals with disabilities (PWD), and mobility plays a crucial role in facilitating this participation. According to the 2022 Canadian Survey on Disability (https://www150.statcan.gc.ca/n1/daily-quotidien/231201/dq231201b-eng.htm, accessed on 29 March 2026) (CSD), approximately 8.0 million Canadians aged 15 and older live with one or more physical or mental disabilities, representing 27% of the population. Among this group, nearly 39% experience motor disabilities that hinder their mobility, making mobility disabilities the third most common type of disability in Canada. In 2022, it was reported that 60% of individuals with milder disabilities faced significant obstacles in accessing both indoor and outdoor public spaces [1]. These barriers hindered their ability to fully participate in activities and engage with their communities. Ban Ki-moon, the United Nations Secretary-General, on the International Day of Persons with Disabilities on 3 December 2012, stated, “Persons with disabilities have a profoundly positive impact on society, and their contributions can be enhanced even further when we eliminate the barriers that hinder their participation.”.

To foster an environment that promotes mobility and social participation of these individuals, it is essential to improve the accessibility of the built environment [2,3,4]. This is particularly important for public buildings and communal spaces, where enhancing accessibility can make a significant difference. By modifying physical structures, such as adding ramps, installing wider doorways, improving signage, and ensuring accessible restrooms, we can create an inclusive environment that encourages participation and interaction in everyday activities [5,6]. These adjustments are advantageous for individuals with mobility challenges and foster a more inclusive and equitable community for all. However, simply implementing these changes may not be sufficient to encourage people with disabilities to participate, as they often lack information regarding accessible routes and locations and guidance on how to reach them. By providing clear and comprehensive information about accessible routes and places adapted to their profile, we can encourage their greater participation in society and improve their quality of life [7].

During the past decades, extensive research has been conducted on the mobility of PWD and the accessibility assessment of urban environments [8,9,10,11,12,13,14,15]. However, most of these studies collected their data manually, which is very time-consuming, slow, and expensive [16,17,18,19,20]. As a result, many cities’ spatial databases lack detailed and up-to-date data on the accessibility of their pedestrian networks. While popular navigation applications such as Google Maps and Maps (Apple) are largely used for car and pedestrian navigation at the city scale, they often fall short of addressing the accessibility issues for mobility and navigation of those with disabilities. Recent efforts in the development of more adapted routing solutions (e.g., AXSMap (https://www.axsmap.com/, accessed on 29 March 2026), Wheelmap (https://wheelmap.org/, accessed on 29 March 2026), or AccessNow (https://map.accessnow.com/, accessed on 29 March 2026) and research [16,20,21,22] have concentrated efforts on elements, including sidewalks, ramps, curb cuts, and street intersections. However, their routing is still fragmented and does not include information on the interface between the indoor and outdoor environments. This highlights the urgent need for more inclusive solutions that integrate detailed information on the environment, enabling any user to plan and navigate adapted routes for their needs.

To address these limitations, this paper proposes a comprehensive and automatic method based on a Virtual Robotic approach for the characterization of pedestrian networks for accessibility assessment using semantically segmented 3D point clouds. The novelty of this research resides in the integration of a Tangent Bug navigation algorithm with a triangular virtual robot model that accurately reflects the width of a standard wheelchair. This approach facilitates automated, scalable characterization of pedestrian pathways within urban environments, eliminating the need for a manual process of the data for accessibility assessment. Unlike existing methodologies that either extract a limited set of features or depend on labor-intensive measurements, the proposed method is not only applicable to the formally documented network in city databases, but also characterizes the linkage between the entrance and main sidewalk, which are mostly ignored in the current navigation tools. To gain a clearer understanding of how and what to measure for each feature related to environmental factors, we utilize the Measure of Environmental Accessibility (MEA) [23]. In the MEA, each pedestrian network component is characterized based on its specific features, such as surface quality, path width, cross slope, running slope, etc. The data extracted on these features can be ultimately used for accessibility assessment on the whole network, which can be ultimately used for personalized accessible route recommendations for people with disabilities.

This study is structured as follows. Section 2 presents a comprehensive review of the relevant literature to establish the theoretical background and identify existing research gaps. Section 3 describes the proposed methodology, detailing the data, models, and analytical procedures employed in this study. Section 4 introduces the case study used to validate the approach, followed by Section 5, which reports and discusses the results. Finally, Section 6 concludes the study by summarizing the main findings and outlining directions for future research.

## 2. Literature Review

Data collection for the characterization and accessibility assessment of the pedestrian network has undergone a significant transformation from labor-intensive manual approaches to increasingly automated methodologies [24]. Traditionally, field surveys conducted by trained personnel documented physical characteristics of sidewalks, curb cuts, slopes, and other environmental features using measuring tools, GPS devices, and standardized checklists [8,18,20,25,26]. While accurate, these methods were inherently time-consuming, costly, and difficult to scale across large urban areas [21,27,28,29].

The proliferation of mobile sensing technologies—including smartphone sensors, wearable devices, and vehicle-mounted equipment—has catalyzed a shift toward automated data collection [30,31]. Contemporary methods increasingly leverage computer vision algorithms applied to street-level imagery, LiDAR point clouds, and crowdsourced platforms to extract accessibility-related features with minimal human intervention [32,33,34,35], enabling more comprehensive and frequent updates to pedestrian network databases.

This shift from manual to automatic data collection not only solves the scalability and timeliness issues of traditional methods but also allows more thorough and frequent updates to pedestrian network databases, supporting real-time accessibility assessments and more inclusive urban planning practices. Despite these advances, a thorough review of the current literature highlights several ongoing gaps and methodological issues. First, most automated methods focus on extracting only a limited set of features—such as sidewalk material [36], surface deficiencies [37], or centerline geometry [38]—without providing a holistic characterization of the pedestrian environment as required by comprehensive accessibility frameworks like the MEA [23]. Second, many studies focus solely on the sidewalk itself, neglecting important transitional zones between building entrances and the pedestrian network, including landing areas, ramps, and intermediate pathways, which are often the toughest for wheelchair users [17,20]. Third, although deep learning segmentation shows promise in classifying urban features, few studies go further to derive key accessibility metrics—like running slope, cross slope, surface roughness, and effective path width—from these segmented outputs [32,39]. Additionally, current automated solutions usually require manual input for setting origin and destination points, which reduces their potential for city-wide use [16,20]. Lastly, none of the reviewed approaches simulate the physical constraints of a real wheelchair during the characterization process, meaning the data collected may not accurately reflect the actual navigability challenges faced by users. These combined limitations highlight the need for a comprehensive approach that can simultaneously extract multiple accessibility parameters across different pedestrian network components while considering the real-world size and constraints of mobility devices. Furthermore, some of these studies’ methods leverage Artificial Intelligence, including deep learning approaches, to extract objects or segments as a data preparation step [32,37,39]. However, the segmented data resulting from this process needs further assessment so it can be used for the accessibility assessment of the network. In other words, we not only need to distinguish between a sidewalk and a vegetation zone, but also, we need to know what the quality of the surface is or what the maximum crosswalk slope is. For instance, vectorization methods can be used to extract information on the width, slope or the centerline of a sidewalk; however, the method is computationally costly and remains somewhat limited in extracting all the necessary data on the features of a given pedestrian network component, such as [35] sidewalk [32,40] sidewalk material [36] tactile [41] width, cross slope, and grade [39] or just the sidewalk center line and its length. Therefore, we aim to develop a novel approach based on robot navigation to characterize and extract the most critical features of various elements, including steps, parking lots, entrance areas, and sidewalks, from a segmented 3D point cloud.

Given the limitations of purely vision-based and vectorization approaches for comprehensive pedestrian network characterization, an alternative paradigm emerges from the field of robotics. Rather than attempting to extract accessibility features through post-processing of segmented data, robotic navigation algorithms offer the possibility of traversing the environment—whether physical or virtual—and collecting multiple parameters simultaneously along the traveled path. This shift in perspective, from static feature extraction to dynamic environment exploration, motivates the integration of path planning techniques into the accessibility assessment pipeline.

In recent years, robotic path planning methods have demonstrated interesting advantages for the assessment of suitable paths for robotic navigation. Mobile robot path planning involves developing a route that minimizes distance and time while avoiding collisions, enabling a mobile robot to travel from its starting point to its destination autonomously [42]. Based on the literature review, robotic path planning can be classified into two broad categories: path planning within a known environment and path planning within an unknown environment. In an unfamiliar environment, the robot starts without maps, relying only on input from its sensors, such as a camera or LiDAR [43]. This category encompasses various bug algorithms for robot navigation and obstacle avoidance. Key members are Bug1 and Bug2 [44], ALG1 and ALG2 [45], the DistBug [46] and Tangent Bug [47] algorithms. For instance, the Tangent Bug algorithm only requires local data and some global awareness, resulting in lower computational demands [48]. In contrast, global path planning algorithms depend on comprehensive environmental maps (e.g., A*). In such situations, path planning relies on the information available on the network and its connectivity information through the navigation environment.

The Tangent Bug algorithm converges globally, effectively sidestepping issues with local minima. It guarantees that the robot will reach its target if a path is available, even in complex environments featuring concave shapes [47]. In the following section, we describe the proposed virtual robotic approach that directly addresses all five identified gaps by simultaneously extracting the most critical accessibility features—including slope, width, surface type, step height, and obstacle clearance—across sidewalks, ramps, entrance areas, and parking lots from a semantically segmented 3D LiDAR point cloud.

## 3. Methodology

**Figure 1:**
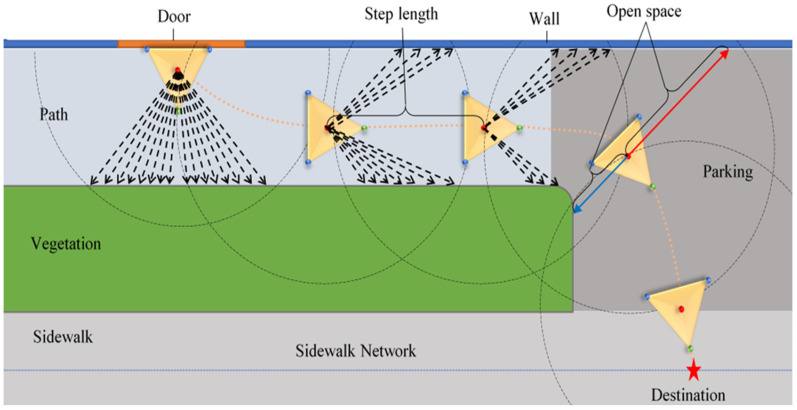
Schematic representation of the virtual robot’s navigation trajectory from building entrance to sidewalk destination, demonstrating the Tangent Bug algorithm’s alternating goal-seeking (orange dashed line) and wall-following behaviors (black dashed arrows). At each step, the triangular robot measures step length, left and right open space (blue and red lines), and terrain class within its sensor Range of View (dashed circles), navigating across path, vegetation, sidewalk, and parking surface classes. Here, the blue dashed line indicates the sidewalk network, and the star marks the robot’s destination.

Here, we propose an automated method for the characterization of pedestrian networks for accessibility assessment using a triangular virtual robot (Figure 1). The virtual robot is shaped as an equilateral triangle because it is the simplest polygon that defines a plane in 3D space, allowing direct calculation of surface normal vectors via the cross product of its edges. Its three vertices act as ground contact points—a geometric analogy to the two rear wheels and front caster of a wheelchair—providing the minimum number of non-collinear contact points required to uniquely define a plane in 3D space, thereby enabling computation of surface normal vectors. It should be noted that this triangular configuration is solely for computational purposes and does not aim to represent an exact geometric model of a wheelchair nor its kinematic model and locomotion; hence, parameters such as turning radius and steering dynamics are outside the scope of this characterization-focused framework.

The centroid of the triangular virtual robot provides a reliable reference point for tracking trajectories and measuring lateral clearance, while the symmetric shape ensures unbiased sensing in all directions. Additionally, the triangle’s side length can be adjusted to match a typical wheelchair footprint, making sure that parameters such as path width and obstacle clearance reflect actual navigability constraints. This robot navigates through a carefully classified 3D LiDAR point cloud, operating within a 3D environment where each point is pre-assigned to specific categories such as sidewalks, vegetation, buildings, and other relevant object classes.

As the robot embarks on its journey from a given start point to its intended destination, it performs simultaneous analysis of its path to ensure its safe movement on the network. Throughout its navigation, the robot faces various constraints, including obstacles, the traversability of different terrains, and its own movement radius. This information is crucial in determining its ability to navigate effectively and safely within the pedestrian network.

The design parameters of the virtual robot and the selection of extracted features are grounded in established accessibility standards, including the Americans with Disabilities Act (ADA) Accessibility Guidelines and the International Building Code (IBC), as well as the MEA framework. The robot’s side length reflects the footprint of a standard manual wheelchair, consistent with the ADA minimum clear floor space of 0.91 m (36 inches). Running slope measurements are referenced against the ADA maximum of 5% (1:20), cross-slope against the 2% (1:50) limit under ADA §403.3, step height against the §303 maximum of 6.4 mm, ramp slope against the §405.2 maximum of 8.33% (1:12), and path width against the ADA minimums of 0.91 m for single passage and 1.52 m (60 inches) for passing space. Importantly, however, the method does not evaluate features as binary pass/fail values against fixed thresholds. Instead, it records continuous, raw measurements of the environment as it physically exists, enabling personalized accessibility assessment tailored to individual user profiles and mobility constraints. This is a deliberate design choice, recognizing that universal standards define minimum compliance baselines but cannot capture the full diversity of disability experiences—a route that satisfies ADA requirements may still pose genuine barriers for certain users, while one that marginally exceeds a threshold may remain navigable for many others. The proposed framework, therefore, produces a rich, flexible dataset that can be interpreted against any standard, clinical guideline, or user-specific constraint, positioning it as a tool for inclusive and personalized urban accessibility evaluation.

Figure 1 illustrates the navigation process of the triangular virtual robot as it moves through a 3D segmented point cloud. The figure demonstrates the robot’s path, highlighting its interactions with different objects/obstacles in the area. One can observe how the robot employs its sensors to detect and respond to changes in the environment (obstacles, navigable areas), enabling it to make adjustments to its route for efficient navigation.

To accomplish the objectives of this study, the proposed methodology is structured around two main components: (a) the Navigation Component and (b) the Data Collection and Characterization Component. The first component involves the development and implementation of a navigation algorithm that will facilitate efficient movement in the virtual classified 3D point cloud. The second component focuses on collecting data and characterizing the environment, providing essential insights and a comprehensive understanding of the context in which navigation occurs.

### 3.1. Navigation Component: Tangent Bug Algorithm

We propose to adapt a Tangent Bug algorithm for autonomous navigation through a virtual environment composed of a classified 3D LiDAR point cloud. The Tangent Bug algorithm is a range-sensor-based navigation algorithm designed for robot path planning in unknown environments [48]. It operates by alternating between two fundamental behaviors: goal-seeking and wall-following (boundary-following) [48,49,50]. Here, the wall can be the border or boundary of any group of points that is not in navigable classes, such as vegetation, roads, and buildings. Navigable classes are those classes that the robot can move on top of, such as Sidewalk, Ramps, Parking lot, Crosswalk, and Steps. In this study, the group includes sidewalks, paths, and parking lots. Our implementation extends the traditional 2D Tangent Bug to operate in complex 3D real-world environments, overcoming limitations such as the lack of point clouds due to occlusion.

While the core logic of the Tangent Bug algorithm—alternating between goal-seeking and border/wall-following modes—is well established, our implementation introduces three key adaptations for 3D LiDAR point cloud environments. First, the traditional 2D occupancy grid is replaced by a 3D semantic grid constructed directly from classified point cloud data, where each cell’s navigability is determined by the semantic class of its constituent points rather than binary obstacle presence. Second, the wall boundary is defined not by physical walls but by the semantic boundary between navigable classes (sidewalks, ramps, paths) and non-navigable classes (vegetation, buildings, roads), enabling semantically aware obstacle avoidance. Third, the implementation incorporates explicit handling of occlusion-induced point cloud gaps, preventing false free-space detection in regions where points are absent due to sensor shadowing rather than genuine navigable space.

**Figure 2:**
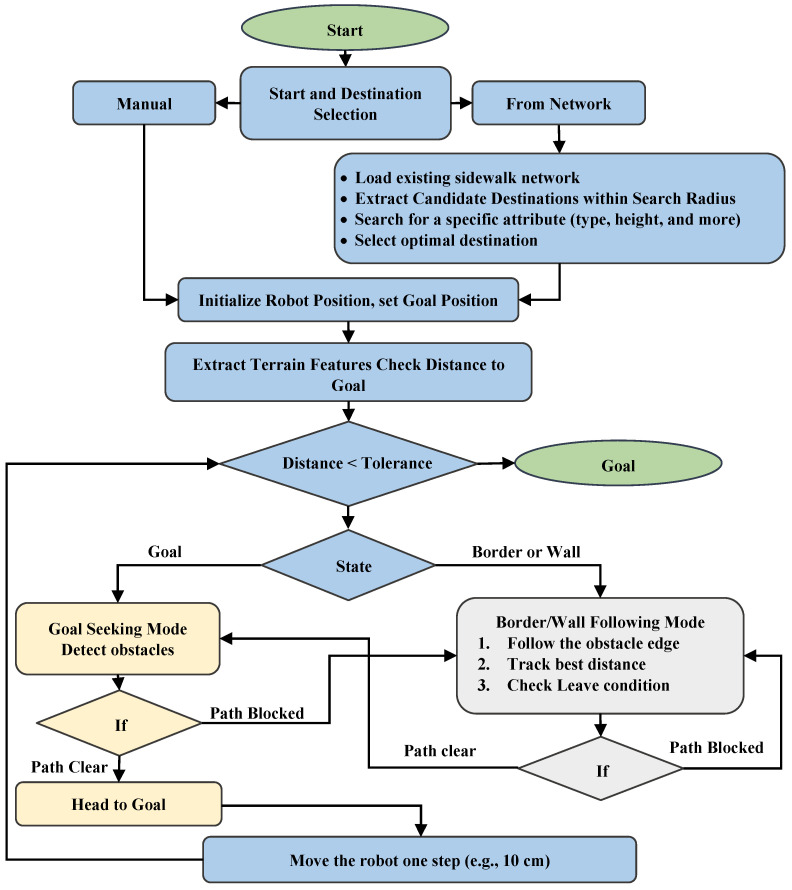
Flowchart of the Tangent Bug algorithm for pedestrian path characterization, illustrating start and destination selection modes, goal-seeking, and border/wall-following navigation states. Here, the blue color shows the main steps of the algorithm, the yellow color shows the goal-seeking steps, and the gray color shows the wall-seeking steps.

Figure 2 illustrates all the steps that the Tangent bug algorithm must follow to accomplish its objective. Each stage is clearly defined, showcasing the systematic approach the algorithm takes to navigate its environment and achieve the desired outcome. The algorithm begins by receiving a classified point cloud, which provides a structured semantic representation of the environment, along with the coordinates for the starting point (e.g., building entrance or door) and the destination, which may be specified either as a discrete point coordinate or derived from an existing sidewalk network, as shown in Figure 1. Initially, the algorithm evaluates the destination input type. If a specific point coordinate is provided, the system proceeds directly to the navigation phase (manual mode, as illustrated in Figure 2. If no destination is set, the algorithm starts by loading the sidewalk network and indexing segments within a 3–15 m radius, depending on urban layout. It then filters candidates based on attributes, such as segments near parking spaces with consistent elevation to the road, which are pre-classified in our database. Upon identifying a suitable segment, the destination point is automatically defined at the centroid of the selected segment. Then the robot is positioned at the origin (door), and as it embarks on its journey towards the destination, its location is continuously updated based on the point cloud data that reflects the terrain and obstacles in its vicinity while taking into account the actual dimensions of a mobility aide device, such as a wheelchair. This dynamic positioning allows the robot to navigate efficiently through its surroundings, adapting its movement to the objects and elements represented in the point cloud. Next, it evaluates the terrain features, such as the surface type and its Euclidean distance from the destination. To expedite the process and reduce computational costs, the algorithm employs a grid mapping process. Grid mapping, commonly referred to as occupancy grid mapping, is a fundamental technique in robotics that facilitates the representation and comprehension of spatial environments [51]. This method discretizes continuous space into a structured grid format, where each cell conveys information about the occupancy status of that specific region. The core principle involves partitioning the robot’s environment into a limited number of uniform cells, typically in square or rectangular shapes, resulting in a two-dimensional grid. Each cell represents a small segment of the real world and contains probabilistic data indicating whether that space is occupied by obstacles or available for navigation [52,53]. The algorithm utilizes a distance-finding function to map the robot’s surrounding environment and aims to minimize the path to the target. The map is commonly known as the local tangent graph, as it is specifically designed to show within the sensor’s Range of View (ROV).

**Figure 3:**
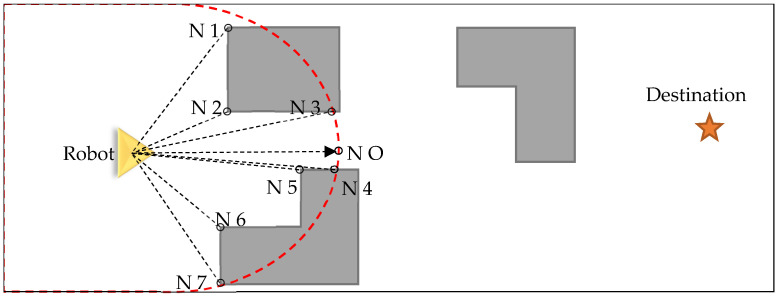
Schematic of the local tangent graph constructed by the virtual robot within its sensor Range of View (ROV, red dashed circle). Barrier nodes N1–N7 mark the angular extremities of detected non-navigable obstacles (black dashed lines), while NO denotes the nearest point along the direct robot-to-destination axis (black dashed arrow). This local graph drives the algorithm’s state decision—goal-seeking or border/wall-following—at each navigation step.

Figure 3 provides a visual representation of how this localized graph is constructed and utilized to assist the robot in navigating its route toward the designated destination point. Creating a local graph requires collecting information from the state functions: the barrier determination function and the free space function. The barrier determination function measures the distance from the robot’s current position to the nearest non-navigable point (e.g., vegetation, buildings, or walls) along each angular direction θ within the sensor’s Range of View (ROV). Specifically, for every radial direction θ emanating from the robot, the function identifies the closest point that does not belong to a navigable class and records its distance, thereby constructing a polar distance profile of the surrounding obstacles—as illustrated by the barrier nodes N1 through N7 in Figure 3. In contrast, the free space (points in navigable classes) function represents the available free space in front of the robot.

The state resulting from the state function includes two modes:

1. The target is visible, and a node name T is created on it if d (R, G) ≤ D, where R represents the robot position, G denotes the goal or destination position, and D indicates the maximum of the sensor’s ROV.
2. If the RT line does not intersect with any point that is not navigable, it indicates that there are no obstacles in its path. Therefore, it will enter the goal-seeking state; otherwise, it will follow the border/wall, which corresponds to the boundary of surrounding non-navigable classes.

If the robot’s distance from its destination exceeds the tolerance, it checks if it can reach the goal directly or if there is a barrier. If it can reach directly, it moves forward one step; if not, it uses wall-following to navigate around obstacles, tracing boundaries while tracking the distance. When it finds a boundary point with a clear path to the goal and a closer distance than before, it exits wall-following and resumes direct navigation.

**Figure 4:**
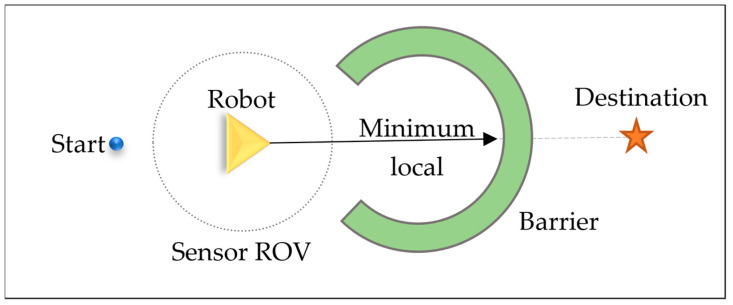
Example of a local minimum situation encountered by the virtual robot during navigation. The robot, positioned between the Start point and the Destination, faces a concave C-shaped barrier whose opening aligns with the direct path to the goal. Since the entire barrier fits within the sensor’s ROV, the robot perceives no clear tangent node to escape, causing it to stall at the local minimum point along the direct trajectory. Here, the black arrow shows the straight path to the goal or destination.

There are situations where the robot can get trapped in a minimum local position (Figure 4). This can occur when it encounters a situation where it believes it has found the optimal path [43]. Still, it gets stuck in a local position, surrounded by obstacles or objects. However, getting trapped in a local minimum is highly dependent on the sensor’s ROV. Increasing the field of view prevents the robot from becoming trapped in a small local minimum; however, it takes more time to process the entire surrounding to void such situations, the wall-following function is designed to reduce the likelihood of the robot becoming trapped in a local minimum, Figure 4 shows the local minimum, sensor ROV, and the positions of the start and destination points. By implementing this function, the robot gains the ability to explore alternative paths and solutions, enhancing its overall efficiency and problem-solving capabilities.

### 3.2. Data Collection and Characterization

To systematically investigate and characterize the navigational path defined by the semantic segments of the 3D LiDAR point cloud identified as a part of a pedestrian network, we developed a virtual robot designed in the form of an equilateral triangle. This unique shape consists of three equal-length sides and angles, providing distinct vertices and a centroid that serve as essential reference points during our experimentation. By strategically using these vertices and the centroid, we were able to collect precise data on the robot’s movement and trajectory as it navigated through different environments. These data include centroid coordinates, step length, open space left, open space right, path width, surface normal, slope, cross-slope, and surface type. Along with these important factors, we calculated additional ones, such as centerline offset, encountering the step pads, and step height. To provide a more precise understanding, we will detail each feature and outline the methods we used for their calculations.

Step length: The step length quantifies the spatial displacement between consecutive robot configurations by measuring the Euclidean distance between the centroids of the triangles. Figure 1 represents the step length.

step length = ||ci − c (i − 1) ||_2_ (1)

where ci = (v1 + v2 + v3)/3 is the centroid position at step I, and v1, v2, and v3 are the vertices of the triangular robot

Open space left and right: Lateral navigable space quantification through perpendicular ray-casting from the robot’s current position, terminating at the boundary between navigable and non-navigable terrain classifications. Figure 4 illustrates how much clear space exists on the left (red) and right (blue) sides of the robot before hitting obstacles.

left = max {d: ∀r ∈ [0, d], p + r · n_⊥ ∈ S_nav}(2)

right = max {d: ∀r ∈ [0, d], p – r · n_⊥ ∈ S_nav}(3)

Here, p indicates the robot’s position, n_⊥ represents the perpendicular vector to its heading defined as [-h_y, h_x, 0], and S_nav refers to the set of navigable surface points. The parameter r is a scalar distance that increments along the perpendicular direction from the robot’s position, while d represents the maximum lateral distance at which all intermediate points between the robot and that distance remain within the navigable surface. In other words, the equations cast a ray perpendicularly to the left and right of the robot and measure how far it extends before encountering a non-navigable boundary.

Path width: path width is determined by the sum of the left and right clear spaces. This combined evaluation allowed us to calculate the overall width of the path accurately.

Surface Normal: The direction that points straight up from the slanted ground surface beneath the robot represents the unit normal vector, which characterizes the local surface orientation. This vector is calculated using the cross product of the edge vectors of the triangle formed by the robot’s support points.

(4) $$\hat{n}=(e_{1}\times e_{2})/{\left||e_{1}\times e_{2}|\right|}_{2}$$

where e_1_ = v_2_ − v_1_ and e_2_ = v_3_ − v_1_ represent the first and second edge vectors, respectively, × denotes the cross product, and the constraint $\hat{n}\_z>0$ ensures the normal points upward.

Slope: refers to the steepness of a surface, ranging from flat (0°) to vertical (90°). It indicates the angular deviation of the local surface normal from the direction of gravity, highlighting the steepest gradient direction.

(5) $$\theta \_\mathrm{slope}=\arccos\;(\hat{n}\cdot \hat{z})$$

where $\hat{n}$ denotes the surface normal vector, ẑ = [0, 0, 1] represents the vertical reference, and the value of θ_slope ∈ [0°, 90°].

Cross slope: refers to the degree of sideways tilt of the ground in relation to the robot’s forward direction. It quantifies the lateral surface inclination that is perpendicular to the robot’s heading, describing the roll characteristics of the terrain compared to the intended path. The positive result is indicated with the right side facing down, while the negative result is shown with the left side facing down.

(6) $$\theta \_\mathrm{cross}=\arcsin\;(\hat{n}\cdot ĥ\_⊥)$$

where ĥ_⊥ = [-h_y, h_x, 0] denotes the vector perpendicular to the heading direction, and θ_cross ∈ [−90°, 90°].

Surface Class: This refers to the type of surface on which the robot is standing, such as a sidewalk, path, or parking lot. This classification is based on semantic labels derived from LiDAR point clouds. It utilizes nearest-neighbor spatial queries within a specified tolerance radius for classifying discrete terrain.

In the final stage of the characterization process, the collected step-by-step measurements are combined and attached as attributes to their respective pedestrian network segments. As the robot moves from origin to destination, consecutive centroid positions are connected to create a linear geometry representing the traveled path. Each segment—such as a sidewalk, ramp, building-to-sidewalk linkage, or any other pedestrian network component—is then enhanced with summarized attribute values derived from individual step measurements, including average path width, maximum slope, mean cross slope, surface type, and overall segment length. When consecutive steps have similar attribute values within a predefined threshold, adjacent micro-segments are merged into larger, homogeneous segments, reducing data redundancy while maintaining meaningful surface transitions. This process ultimately produces a fully attributed pedestrian network in vector line format, where each segment carries the quantitative accessibility parameters needed for accessibility assessment frameworks and, ultimately, for integration into navigation tools for adapted route planning for people with mobility impairments.

## 4. Case Study

To effectively address the authentic challenges encountered by wheelchair users, our virtual environment is constructed utilizing an authentic point cloud. The point cloud dataset was meticulously collected from the area of Victoriaville in Quebec, Canada, by the dedicated team at Trifid Group (https://www.groupetrifide.com/, accessed on 29 March 2026). The coordinate datum utilizes the NAD_1983_MTM_7 horizontal coordinate system, along with the NAD_1983 vertical coordinate system. The point cloud has been meticulously annotated using CloudCompare software version 2.12.4, categorizing the data into 35 distinct classes for enhanced analysis and usability. Among these, the navigable categories encompass a variety of essential infrastructure elements, such as sidewalks, ramps, parking lots, bare surfaces, curb cuts, and crosswalks. Each class has been defined to facilitate specific applications, ensuring that users and robots can efficiently identify and interact with different features in the environment. This detailed classification not only improves accessibility and navigability for various pedestrian mobility modes but also aids in urban planning and development.

## 5. Results and Discussion

**Figure 5:**
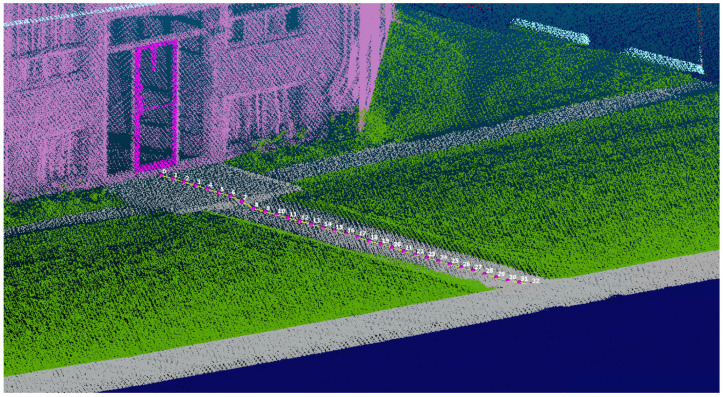
Perspective view of the semantically classified 3D LiDAR point cloud showing the virtual robot’s trajectory (numbered centroid positions in pink/white) navigating from a building entrance (magenta rectangle) through a maneuvering area and path toward the sidewalk network (gray), with vegetation (green), building façade (pink), and road surface (dark blue) visible.

Figure 5 illustrates the results from the characterization performed using the proposed method by defining a virtual robot shaped like an equilateral triangle. Table 1 summarizes a selection of results obtained from our virtual robot’s exploration of a classified point cloud. Following the Measure of Environmental Accessibility framework [23] introduced in Section 1, the virtual robot generates comprehensive data for each discrete movement step, recording 13 primary parameters that directly correspond to the key environmental features defined in the MEA. Specifically, the MEA identifies surface type, path width, running slope, cross slope, and surface condition as critical features for characterizing pedestrian network components such as sidewalks, ramps, pathways, and building access linkages. Our system captures these features through the following recorded parameters: surface class (corresponding to the MEA’s surface type), spaces on the left and right combined as path width (corresponding to the MEA’s path width requirement), longitudinal slope (corresponding to the MEA’s running slope), cross slope (corresponding to the MEA’s cross slope), and surface normal vectors from which surface roughness can be derived (corresponding to the MEA’s surface condition). Additional parameters, such as step detection and step height, address the MEA’s requirements for characterizing vertical transitions, while centerline offset and distance to destination support navigation quality assessment. Centerline offset measures the lateral deviation of a detected path or object from the expected route centerline, indicating how well a navigation system maintains alignment with the intended trajectory. Distance to destination quantifies how accurately the system estimates remaining travel, providing a direct measure of localization and path-planning performance. Together, these two metrics allow evaluators to assess whether a navigation system can reliably guide a user along the correct path and accurately track progress toward a goal.

Table 1 presents the first 10 steps of the trajectory in Figure 5, showing variations in path width from 0.2 m at the entrance (Step 0) to 2.7 m on an open pathway (Steps 1–3), narrowing to 1.2 m on a constrained segment (Steps 8–10). Notably, a steep transition occurs at Step 7 (10.56°), indicating a potential accessibility barrier. Each step is assigned a unique identifier for tracking. The robot’s location is recorded as the centroid of its triangular footprint in a 3D coordinate system (centroid x, centroid y, centroid z). Measurements in Table 1 are rounded to two decimal places, with the original CSV file retaining five. Figure 5 visually represents the trajectory by tracking the centroid coordinates, highlighting essential features extracted during navigation for comparison against MEA thresholds.

While the system successfully records 13 parameters per navigation step, a formal quantitative validation of the extracted measurements against ground truth field data still needs to be performed. Preliminary qualitative assessments suggest that the system captures meaningful variations consistent with the physical environment. When interpreting the extracted values against established accessibility standards—specifically the ADA maximum running slope of 5% (2.86°), maximum cross-slope of 2% (1.15°), and minimum path width of 0.91 m—it becomes possible to understand the practical accessibility implications of the recorded parameters. For example, the steep slope detected at Step 7 (10.56°, approximately 18.7%) significantly exceeds the ADA maximum for accessible routes, indicating a major obstacle for wheelchair users and possibly representing a step, ramp, or sudden terrain change in the point cloud. Likewise, the narrow path width of 0.2 m recorded at Step 0 indicates a tight building entrance zone, or that the first step is very close to the entrance—an area the robot could not measure accurately—which falls below the ADA minimum clear width and would prevent independent wheelchair passage without structural changes. Conversely, path widths of 2.6–2.7 m recorded at Steps 1–3 comfortably surpass accessibility requirements, showing sufficient lateral space for wheelchair navigation along the open pathway segment.

Regarding the outlier observed at Step 4, where the path width reaches 11.5 m and the space on the left extends to 10 m, this value is not an error but an accurate reflection of the physical scene. At this location, the robot is positioned between a sidewalk intersection, and the algorithm correctly measures the full lateral navigable space spanning both sidewalks. This configuration—where the robot traverses an open transitional zone flanked by two sidewalk edges—produces a legitimately wide path width measurement that accurately represents the spatial geometry at that step. This underscores the importance of interpreting extracted parameters in their spatial context rather than applying uniform thresholds globally.

The system calculates the Euclidean distance traveled between consecutive positions (step length), which typically equals the configured step size of 0.3 m during normal navigation but may vary during obstacle avoidance or step climbing maneuvers. This step size was selected based on the minimum tread depth specified in building standards: both the International Building Code (IBC) [54] and the ADA [55] require a minimum tread depth of 11 inches (28 cm). By setting the step size to 30 cm—approximately 2 cm larger than the strictest minimum tread depth—we ensure that at least one robot position falls on each individual stair tread during step detection and climbing sequences. This guarantees that no step is skipped during characterization. However, varying step lengths are possible. A reduction in this distance would provide a more accurate representation of the terrain’s shape; however, it would also require additional time to complete the process. The system continuously monitors the open navigable space perpendicular to the robot’s heading direction, recording the distances to the nearest obstacles on both the left (space on left) and right (space on right) sides. The sum of these measurements yields the total path width at the current position. These parameters directly influence the navigation algorithm’s decisions, particularly when the path width approaches the minimum passage threshold of 0.8 m, below which the robot must seek alternative routes. The space on the left and right shows the open area on either side of the robot, considering the direction of its trajectory. Surface geometry is characterized through multiple parameters derived from the robot’s support triangle. The vertical component of the surface normal vector (Normal z) indicates surface flatness, with values approaching 1.0 representing horizontal surfaces. The system calculates both longitudinal slope (slope) and lateral cross-slope (cross-slope) angles in degrees, providing essential information for stability assessment and motion planning. Surface classification (class) is extracted from the point cloud data, with navigable surfaces identified by a predefined class. The robot’s progress toward its destination is continuously monitored through the measurement of the straight-line distance to the goal. A key factor in our approach is the centerline offset parameter, which quantifies the robot’s lateral deviation from the ideal centered path. This deviation is calculated by assessing the robot’s position relative to non-navigable points located on its left and right sides. This signed value indicates displacement toward the left (positive) or right (negative) side of the navigable corridor, enabling the control system to maintain optimal positioning equidistant from obstacles. The system incorporates specialized parameters for vertical terrain discontinuities. The binary step detected flag indicates the presence of a navigable step within the robot’s sensor range, while step height quantifies the vertical displacement in meters. Positive values indicate ascending steps, negative values indicate descending steps, and the system enforces a maximum navigable height threshold of 0.15 m for robot movement. When no step is present, both parameters default to FALSE and 0.0, respectively.

**Figure 6:**
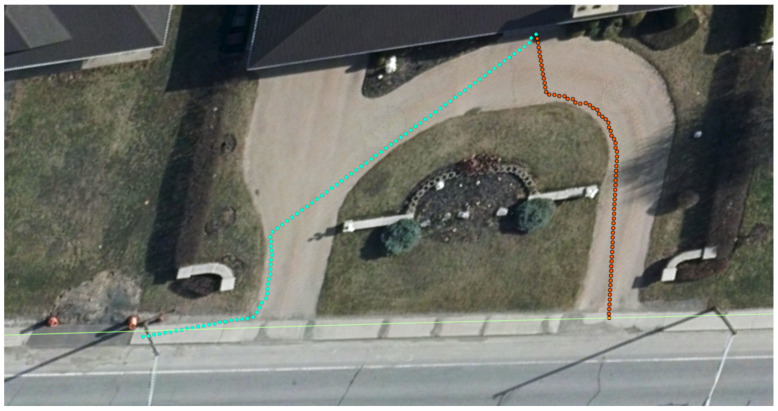
Aerial view of two characterized building-to-sidewalk trajectories overlaid on orthophoto imagery, showing the cyan path (longer, curved route navigating around a central landscape feature) and the orange path (shorter, direct route along the right side of the property) from building entrances to the sidewalk network.

**Figure 7:**
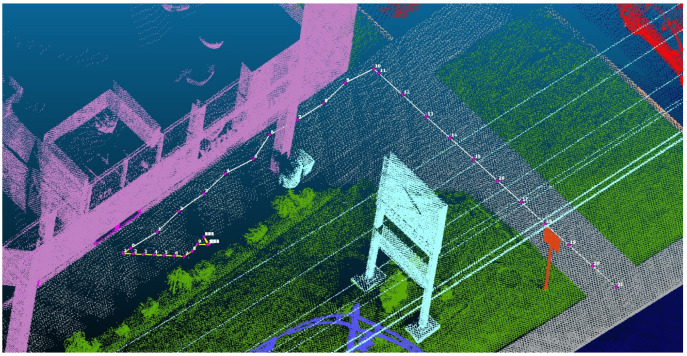
Perspective view of the semantically classified 3D LiDAR point cloud illustrating the impact of grid resolution and step length on the virtual robot’s characterized trajectory. Two navigation scenarios are shown: a yellow line and a pink dotted path using a finer grid resolution of 10 cm with a step length of 30 cm (number shows the steps number), and a white line path using a coarser grid resolution of 25 cm with a step length of 100 cm. Both trajectories navigate from the same building entrance toward the sidewalk network, demonstrating how parameter selection influences path detail and spatial precision of the characterization output.

In Figure 6, the robot’s ability to maintain a safe distance from obstacles is illustrated. It effectively identifies its destination (red dots) from the sidewalk network. It navigates around objects that are not part of its navigable path, ensuring that it avoids potential collisions and creates an accessible path. Figure 5, Figure 6 and Figure 7 represent the robot’s path and characterization, which allows it to precisely measure the proximity of surrounding objects and adjust its trajectory accordingly.

Due to the inherent challenges associated with point clouds, such as the presence of missing data caused by occlusion, it is essential to define a no-point space in the point cloud used as the virtual environment for robot navigation. In this project, we establish this no-point space through a grid-based mapping system with a 10 cm grid value. This mapping system helps the algorithm find places where there is no point. The resolution of the grid plays a crucial role in system performance. Smaller grid values (e.g., 5 cm cells) offer higher spatial resolution and enhance the precision of no-point and obstacle boundary detection, facilitating more refined navigation control. However, this increased precision comes with a trade-off in terms of computational overhead, memory usage, and processing time, as the number of cells grows exponentially. This can result in less accurate obstacle representation and suboptimal path planning, especially in environments with narrow passages or small obstacles that may not be effectively captured at coarser resolutions. Figure 7 illustrates the effect of the no-point area on robot navigation across various grid sizes. The yellow line in Figure 7 represents a scenario with a 10 cm grid size and a 30 cm step length, demonstrating how the robot becomes unable to navigate between a wall and a no-point area, ultimately getting stuck in a local minimum. In contrast, the white line indicates that, by increasing the grid size to 25 cm and the step length to 100 cm, the robot was able to navigate around obstacles and successfully find its path to the destination.

### 5.1. Sensitivity and Performance of the Algorithm

The three key parameters governing the virtual robot’s performance are the occupancy grid resolution, the robot’s step size, and the sensor ROV. These parameters interact to determine three performance dimensions: navigation success rate (the proportion of trajectories that reach the destination without becoming trapped in local minima), feature extraction accuracy (the reliability of slope, cross slope, and width measurements relative to ground truth), and computational efficiency (processing time per meter of characterized path). Regarding grid resolution, it is important to recognize that the choice of grid size is closely linked to the underlying point cloud density: a grid cell size smaller than the average distance between points can lead to underpopulated cells and unreliable occupancy classification. Finer grids (e.g., 5–10 cm) provide greater spatial accuracy in detecting obstacle boundaries and calculating surface normals, which directly enhances the accuracy of slope and width measurements. However, increasing resolution causes the number of grid cells to grow quadratically (a 5 cm grid results in four times as many cells as a 10 cm grid), leading to proportionally higher memory use and longer processing times. Conversely, coarser grids (e.g., 25–30 cm) reduce computational overhead but risk overlooking narrow obstacles and small terrain features, which is particularly problematic in confined scenarios such as narrow passages or building entrance areas where obstacles may be only 10–20 cm wide. In general, we can utilize finer grids in areas with higher point density, such as those near the mobile LiDAR sensor. Conversely, larger cells can be applied in regions with lower density, like entrances that are farther from the sensor, or in areas where point data is missing due to masking.

Regarding step size, smaller steps (e.g., 10–30 cm) increase the density of measurements per unit length, thereby improving the characterization of rapidly changing terrain features such as ramp transitions and curb cuts; however, they proportionally increase the total number of navigation iterations and associated computations. Larger steps (e.g., 80–100 cm) improve computational throughput but may skip over short terrain transitions and reduce the effective resolution of slope measurements. Based on the observations from our case study and the quantitative verification experiment, we propose the following preliminary scenario-based parameter guidelines: for narrow passages and building entrance linkages, where obstacles are closely spaced and terrain transitions occur over short distances, a fine grid resolution of 10 cm combined with a step size of 30 cm and a moderate ROV is recommended to ensure that small obstacles are detected and that the robot does not become trapped between closely spaced boundaries; for open sidewalks with gradual slope variations and fewer nearby obstacles, a moderate grid resolution of 5–20 cm with a step size of 30–50 cm provides a favorable balance between measurement accuracy and computational cost; for ramp areas, where a running slope changes rapidly over a short distance, a step size of 20–50 cm is preferred with 5 to 10 cm grid resolution to capture the slope gradient with sufficient fidelity.

**Figure 8:**
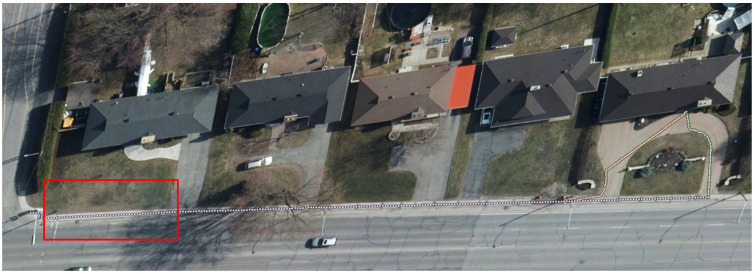
Top-down orthophoto view of multiple virtual robot trajectories (white/pink dotted paths) characterized along a residential street block in Victoriaville, Quebec. Two trajectories navigate from a building entrance to the sidewalk network through the semantically segmented 3D LiDAR point cloud, collecting accessibility parameters, including running slope, cross-slope, lateral clearance, and obstacle proximity. The diversity of path lengths and geometries across properties demonstrates the method’s adaptability to varying urban configurations. The red box represents the area that we use for quality assessment.

While this example was specifically directed toward evaluating the accessibility of the path connecting building entrances to the primary sidewalk infrastructure, the proposed methodology has broader applicability. The virtual robot navigation framework is capable of traversing and characterizing various pedestrian network components, including sidewalks, crosswalks, and curbs, as illustrated in Figure 8. It shows that this method has the capability to characterize all types of paths, even when the start and destination are far from each other. By analyzing the path traced by the robot and creating a detailed attribute table, we can conduct additional calculations to derive more significant insights. This process allows us to combine or merge smaller line segments into larger ones that share the same attributes, based on a predetermined threshold for merging. This approach enhances the overall data representation and facilitates more comprehensive analyses. In addition, we can estimate the roughness of the terrain by analyzing the variations observed with each measured step over a designated distance. For example, if we set a step size of 10 cm or less, we can record the z-normal value for that position along with the vertical value for each step taken. A significant difference between the z-normal and vertical values of two consecutive steps suggests a change in the terrain’s texture or elevation. By continuing this process over a specified distance and aggregating the differences in height and z-normal values, we can effectively assess the roughness of the path taken.

Employing a virtual robot to analyze and characterize a pathway offers numerous significant advantages. One of the key benefits is the ability to extract a diverse range of features that cannot be comprehensively obtained using traditional data extraction techniques, such as centerline extraction. This capability is especially valuable, as we focus on establishing a connection between the entrance and the main sidewalk network, a challenge that is more complex than standard sidewalk characterization. Additionally, utilizing a virtual robot allows us to account for the actual dimensions of a wheelchair, enabling the collection of highly personalized and pertinent data that corresponds to real-world applications. This tailored data collection is essential for ensuring accessibility and usability in public spaces. Furthermore, this approach is notably faster, more cost-effective, and safer compared to conventional data collection methods, whether through manual gathering or the use of physical robots on-site. By opting for a virtual robot, we not only enhance efficiency but also mitigate potential risks and costs associated with on-site data collection.

It is important to note that the raw stepwise data produced by the virtual robot—one set of measurements per 30 cm step—cannot be directly used for accessibility assessment without further post-processing. Several intermediate steps are required to transform these fine-grained measurements into segment-level attributes suitable for integration with accessibility standards and the MEA framework. First, the stepwise records must be grouped into meaningful segments based on transitions in surface class; for example, when the robot crosses from a pathway onto a ramp and then onto a sidewalk, each transition defines the boundary of a distinct pedestrian network component. Second, within each segment, the individual step measurements must be aggregated into representative summary values—such as the average running slope of a ramp, the minimum path width along a sidewalk, or the maximum cross slope within a pathway—as these are the quantities against which accessibility thresholds are evaluated. Third, adjacent micro-segments that share similar attribute values within a predefined tolerance can be merged to reduce data redundancy while preserving meaningful transitions in environmental characteristics. Finally, the resulting attributed segments must be compared against the individual capability for personalized accessibility information or MEA threshold values and relevant accessibility standards (e.g., maximum allowable running slope of 5% for a sidewalk, minimum path width of 0.9 m for wheelchair passage) to classify each segment as accessible, partially accessible, or inaccessible for normative accessibility assessment. This post-processing pipeline bridges the gap between the detailed stepwise data collected during navigation and the segment-level accessibility indicators required by urban planners and navigation applications.

From a computational standpoint, the proposed algorithm operates with a per-path complexity of O(*n*·k), where *n* denotes the number of navigation steps and k represents the local point cloud density within the sensor’s Range of View (ROV) at each step. The dominant computational cost arises from the spatial nearest-neighbor queries performed at each step to construct the local tangent graph and classify navigable boundary conditions. On a standard desktop workstation, a typical 10-m building-to-sidewalk linkage is characterized in a few seconds, demonstrating practical efficiency for individual path processing.

However, overall computational performance is sensitive to several interdependent parameters. As illustrated in Figure 7, grid resolution has a direct impact on both navigation success and processing cost: a finer grid (e.g., 5 cm) yields higher spatial precision but increases memory consumption and processing time, while a coarser grid (e.g., 25 cm) reduces computational overhead at the cost of potentially missing narrow obstacles or subtle terrain features. Similarly, reducing step length improves terrain characterization fidelity but increases the number of steps and associated nearest-neighbor queries per trajectory. A systematic benchmarking study—reporting processing time per meter, peak memory usage, and total operations as functions of point cloud density, grid resolution, ROV, and step length—is identified as a priority for future work. Regarding comparison with existing methods, a direct computational benchmark against point cloud-based vector extraction or traditional 2D Tangent Bug algorithms is not methodologically appropriate: the former produces only geometric attributes without surface classification or obstacle characterization, while the latter was designed for planar navigation and performs no feature extraction. The only functionally equivalent approach is a manual field survey, which the proposed method substantially outperforms in terms of time, cost, safety, and scalability.

### 5.2. Quantitative Comparison of the Obtained Results with the Ground Truth

A sidewalk segment, highlighted in red in Figure 8 and measuring approximately 22.5 m in length, was chosen from the study area. This segment consists of 75 navigation steps (with a step length of 0.30 m and a 5 cm grid) and represents a typical urban sidewalk scenario, featuring varying running slopes, cross slopes, and a consistent width. These characteristics are essential for assessing pedestrian accessibility in real-world conditions. We selected this area due to our lack of access to the site, allowing us to extract all necessary data from the high-resolution point cloud, which we previously utilized for the virtual robot method. Consequently, we chose a straightforward area that facilitates manual measurements of width, slope, and cross slope.

Table 2 summarizes the accuracy metrics computed from the 75-step comparison between the virtual robot’s extracted parameters and the manual data extraction.

Path Width: The data extracted by the virtual robot demonstrates excellent agreement with the manually extracted data for the path width estimation. The Mean Absolute Error (MAE) of 0.027 m and Root Mean Square Error (RMSE) of 0.033 m correspond to a Normalized Root Mean Square Error (NRMSE) of only 2.71%, indicating very high measurement precision. The near-zero Mean Bias Error (MBE = +0.005 m) confirms the absence of systematic over- or underestimation. In practical terms, 93.3% of the 75 steps fall within ±0.05 m of the true value, and 98.7% fall within ±0.10 m. These results confirm that the robot’s lateral clearance sensing and width estimation mechanisms are highly reliable for accessibility assessment applications.

Running Slope: The running slope measurements show good agreement with the ground truth, with an MAE of 0.776° and a near-zero MBE of −0.062°, indicating that the robot captures the overall slope pattern without systematic deviation. The Coefficient of Determination (R^2^) of 0.489 indicates that the virtual robot explains approximately 49% of the variance observed in manual measurements. In terms of agreement, 74.7% of steps fall within ±1.0° and 94.7% within ±2.0° of the manually measured values. The higher NRMSE (66.56%) is attributable to the small absolute range of running slope values in this segment, which amplifies relative error. Errors are concentrated in the first few steps of the segment where the terrain transitions from a ramp to the sidewalk in front of a parking lot (a steep-slope region), where the robot’s triangular footprint averages over a rapidly changing surface gradient.

Cross-Slope: The cross-slope results reveal a strong linear relationship with the ground truth (R^2^ computed from the linear regression confirms consistent directional tracking), indicating that the virtual robot accurately captures relative cross-slope variations along the path. However, a systematic negative bias (MBE = −1.315°) indicates that the robot consistently underestimates cross-slope magnitude compared to manual measurements. This bias is attributed to a fundamental difference in measurement methodology: the manual cross slope is measured perpendicular to the walking direction at each step location using a contact-based inclinometer, while the robot’s triangular footprint computes the cross slope from the surface normal vector of its three contact points, which effectively averages the local surface orientation over a larger area (~0.60 m triangle side). Despite this systematic offset, 93.3% of steps fall within ±2.0°, and strong agreement in relative variation suggests that a calibration correction (e.g., a simple linear bias adjustment) could further improve absolute accuracy in future implementations.

### 5.3. Limitations

Despite the encouraging outcomes, the proposed methodology exhibits several limitations that warrant careful consideration. Firstly, the efficacy of the system is inherently contingent upon the quality of the input semantically segmented point cloud. Any misclassification resulting from deep learning algorithms—such as erroneously categorizing a vegetation area as a sidewalk or misidentifying a ramp as a pathway—directly influences navigation decisions and the precision of the derived values for different parameters. This may result in inaccurate slope calculations, improper assignments of surface types, or navigation through areas that are non-traversable.

Secondly, while the MEA framework delineates a comprehensive array of properties for each element within the pedestrian network, the current implementation is limited to capturing only a subset of these attributes. Specifically, it focuses on features that can be derived from the geometric characteristics of the point cloud, including slope, width, surface type, and step height. Crucial accessibility features such as the presence of handrails, visibility of signage, lighting conditions, quality of surface joints, and tactile indicators cannot be extracted from point cloud geometry alone. Their extraction requires integrating additional data sources, such as street-level imagery or advanced object detection models, and crowdsourcing, to identify or extract this data and combine it with the existing dataset [33,41,56,57].

Thirdly, the system demonstrates sensitivity to the completeness of the point cloud; occlusions that lead to data voids are interpreted as obstacles by the algorithm, which can result in the robot becoming trapped in a local minima or necessitating extraneous detours, as illustrated in Figure 7. While adjustments to grid resolution and step length can alleviate this concern, the optimal configuration of parameters remains dependent on the specific environment and currently requires manual tuning. Further investigations are needed to determine the sensitivity of the proposed algorithm to the step size as well as the grid resolution.

Fourthly, the virtual robot is designed to characterize a single path during each execution, implying that comprehensive mapping of an entire urban area necessitates repeated operational runs from every building entrance to the pedestrian network. This process may become prohibitively time-consuming on a city-wide scale without the implementation of parallel processing capabilities.

Lastly, it is pertinent to note that the point cloud serves as a static representation of the environment, captured at a singular moment in time. Consequently, transient conditions—including parked vehicles, construction sites, snow accumulation, or seasonal vegetation changes—are not taken into account, and the accessibility data extracted may not fully reflect typical or year-round conditions. Addressing this limitation in future work could involve integrating crowdsourced accessibility platforms such as Wheelmap or OpenStreetMap to capture reported dynamic obstacles, or applying change detection algorithms on periodically updated point clouds to trigger re-characterization of affected segments only. The modular, segment-independent architecture of the proposed virtual robot makes it well-suited for such incremental dynamic updates.

## 6. Conclusions

This paper introduces a virtual robotic method for the automated accessibility characterization of pedestrian networks utilizing semantically segmented 3D LiDAR point clouds. The Tangent Bug algorithm, modified for 3D environments and implemented on a triangular virtual robot, successfully extracted 13 parameters, including those aligned with MEA, such as running slope, cross-slope, path width, surface type, and step height, across various surfaces, such as sidewalks, ramps, and building-to-sidewalk connections. Quantitative validation with the ground truth demonstrates high accuracy for path width (MAE = 0.027 m, NRMSE = 2.71%) and reliable slope tracking, with design parameters firmly grounded in ADA and IBC standards. By capturing continuous raw measurements instead of binary compliance values, this approach can support flexible, personalized accessibility assessments that extend beyond universal threshold constraints. The method addresses three significant gaps in existing literature: comprehensive multi-parameter characterization, consideration of transitional zones connecting building entries with the sidewalks, and derivation of values from segmented point clouds. This approach substantially outperforms manual field surveys as well as manual measurements from point clouds in terms of time, cost, and scalability. Key limitations include reliance on the quality of upstream point cloud segmentation, the inability to capture dynamic environmental conditions, and the non-consideration of certain MEA attributes that cannot be derived solely from point cloud geometry. Future work will focus on enhancing the robustness of characterization and segmentation while expanding the range of extracted MEA attributes by integrating complementary data sources. Additionally, we will aim to develop a city-level batch processing framework featuring automated building entrance detection and parallel computing to enable scalable deployment of the proposed algorithm, recognizing huge challenges related to efficient assessment of city-wide LiDAR acquisition and its semantic segmentation.

## Figures and Tables

**Table 1:** The values of the extracted factors during the first 10 steps of Figure 5. All distances are based on meters, and slopes are based on degrees.

Step ID	Centroid X	Centroid Y	Centroid Z	Step Length (m)	Space on the Left (m)	Space on Right (m)	Path Width (m)	Slope (Degree)	Cross Slope (Degree)	Class	Distance to Destination (m)	Centerline Offset (m)	Steps Detected	Step Height (m)
0	193,345.58	5,102,427.04	143.33	0.00	0.1	0.1	0.2	0.87	0.33	1111	9.90	0	FALSE	0
1	193,345.77	5,102,427.27	143.33	0.30	1.3	1.4	2.7	0.38	−0.31	1111	9.60	−0.05	FALSE	0
2	193,345.97	5,102,427.50	143.32	0.30	1.3	1.3	2.6	0.17	0.09	1111	9.30	0	FALSE	0
3	193,346.16	5,102,427.73	143.32	0.30	1.3	1.4	2.7	0.96	0.70	1111	9.00	−0.05	FALSE	0
4	193,346.35	5,102,427.96	143.32	0.30	10	1.5	11.5	0.31	−0.31	1111	8.70	4.25	FALSE	0
5	193,346.54	5,102,428.19	143.31	0.30	1.3	1.5	2.8	1.40	0.00	1111	8.40	−0.1	FALSE	0
6	193,346.74	5,102,428.42	143.31	0.30	1.3	1.3	2.6	4.40	0.25	1111	8.10	0	FALSE	0
7	193,346.93	5,102,428.65	143.28	0.30	0.7	0.6	1.3	10.56	−0.03	1111	7.80	0.05	FALSE	0
8	193,347.12	5,102,428.87	143.22	0.31	0.6	0.6	1.2	0.52	0.52	1111	7.50	0	FALSE	0
9	193,347.32	5,102,429.10	143.22	0.30	0.6	0.6	1.2	0.46	−0.41	1111	7.20	0	FALSE	0
10	193,347.51	5,102,429.33	143.22	0.30	0.6	0.6	1.2	1.03	−0.85	1111	6.90	0	FALSE	0

**Table 2:** Quantitative accuracy metrics for the virtual robot’s extracted parameters compared against manual ground truth measurements over a 22.5 m sidewalk segment (75 steps, step length = 0.30 m).

Parameter	N	MAE	RMSE	MBE (Bias)	NRMSE (%)
Path Width (m)	75	0.027	0.033	+0.005	2.71
Running Slope	75	0.776	0.962	−0.062	66.56
Cross Slope	75	1.315	1.594	−1.315	64.89

## Data Availability

The source code for the virtual robotic navigation algorithm developed in this study is publicly available on GitHub at https://github.com/aliaria/Sidewalk-characterization-with-Virtual-Robot, accessed on 29 March 2026. The raw 3D LiDAR point cloud dataset used for the case study and validation experiments is publicly available on Zenodo at https://doi.org/10.5281/zenodo.19354886. All data and code needed to reproduce the results presented in this paper are accessible through the above repositories.

## Abbreviations

The following abbreviations are used in this manuscript:

CSD	Canadian Survey on Disability
MEA	Measure of Environmental Accessibility
ROV	Range of View
CSV	Comma-Separated Values
LiDAR	Light Detection and Ranging
3D	Three-Dimensional
MAE	Mean Absolute Error
RMSE	Root Mean Square Error
NRMSE	Normalized Root Mean Square Error
MBE	Mean Bias Error
